# Metabolomic Identification of a Novel Pathway of Blood Pressure Regulation Involving Hexadecanedioate

**DOI:** 10.1161/HYPERTENSIONAHA.115.05544

**Published:** 2015-07-08

**Authors:** Cristina Menni, Delyth Graham, Gabi Kastenmüller, Nora H.J. Alharbi, Safaa Md Alsanosi, Martin McBride, Massimo Mangino, Philip Titcombe, So-Youn Shin, Maria Psatha, Thomas Geisendorfer, Anja Huber, Annette Peters, Rui Wang-Sattler, Tao Xu, Mary Julia Brosnan, Jeff Trimmer, Christian Reichel, Robert P. Mohney, Nicole Soranzo, Mark H. Edwards, Cyrus Cooper, Alistair C. Church, Karsten Suhre, Christian Gieger, Anna F. Dominiczak, Tim D. Spector, Sandosh Padmanabhan, Ana M. Valdes

**Affiliations:** From the Department of Twin Research and Genetic Epidemiology, King’s College London, London, United Kingdom (C.M., M.M., M.P., T.D.S., A.M.V.); Institute of Cardiovascular and Medical Sciences, College of Medical, Veterinary and Life Sciences, University of Glasgow, Glasgow, United Kingdom (D.G., N.H.J.A., S.M.A., M.M.B., A.F.D., S.P.); Institute of Bioinformatics and Systems Biology, Helmholtz Zentrum München, Germany (G.K., K.S.); Faculty of Medicine, University of Southampton, Southampton, United Kingdom (P.T., M.H.E., C.C.); Wellcome Trust Sanger Institute Human Genetics, Hinxton, United Kingdom (S.-Y.S., N.S.); MRC Integrative Epidemiology Unit, School of Social and Community Medicine, University of Bristol, Bristol, United Kingdom (S.-Y.S.); Chemical Analytics, Seibersdorf Labor GmbH, Seibersdorf, Austria (T.G., A.H., C.R.); Institute of Epidemiology II, Helmholtz Zentrum München, Germany (A.P., C.G.); Research Unit Molecular Epidemiology, Helmholtz Zentrum München, Germany (A.P., R.W.-S., T.X.); Cardiovascular and Metabolic Diseases, Pfizer Worldwide Research and Development, Cambridge, MA (M.J.B.); Edison Pharmaceuticals, Mountain View, CA (J.T.); Metabolon, Inc, Durham, NC (R.P.M.); Nuffield Department of Orthopaedics, Rheumatology and Musculoskeletal Sciences, University of Oxford, Oxford, United Kingdom (C.C.); Scottish Pulmonary Vascular Unit, Golden jubilee Hospital, Glasgow, United Kingdom (A.C.C.); Department of Physiology and Biophysics, Weill Cornell Medical College in Qatar, Education City, Qatar Foundation, Doha, Qatar (K.S.); and Academic Rheumatology, University of Nottingham, Nottingham, United Kingdom (A.M.V.).

**Keywords:** blood pressure, fatty acid synthases, hypertension, metabolomics, mortality

## Abstract

High blood pressure is a major contributor to the global burden of disease and discovering novel causal pathways of blood pressure regulation has been challenging. We tested blood pressure associations with 280 fasting blood metabolites in 3980 TwinsUK females. Survival analysis for all-cause mortality was performed on significant independent metabolites (*P*<8.9×10^−5^). Replication was conducted in 2 independent cohorts KORA (n=1494) and Hertfordshire (n=1515). Three independent animal experiments were performed to establish causality: (1) blood pressure change after increasing circulating metabolite levels in Wistar–Kyoto rats; (2) circulating metabolite change after salt-induced blood pressure elevation in spontaneously hypertensive stroke-prone rats; and (3) mesenteric artery response to noradrenaline and carbachol in metabolite treated and control rats. Of the15 metabolites that showed an independent significant association with blood pressure, only hexadecanedioate, a dicarboxylic acid, showed concordant association with blood pressure (systolic BP: β [95% confidence interval], 1.31 [0.83–1.78], *P*=6.81×10^−8^; diastolic BP: 0.81 [0.5–1.11], *P*=2.96×10^−7^) and mortality (hazard ratio [95% confidence interval], 1.49 [1.08–2.05]; *P*=0.02) in TwinsUK. The blood pressure association was replicated in KORA and Hertfordshire. In the animal experiments, we showed that oral hexadecanedioate increased both circulating hexadecanedioate and blood pressure in Wistar–Kyoto rats, whereas blood pressure elevation with oral sodium chloride in hypertensive rats did not affect hexadecanedioate levels. Vascular reactivity to noradrenaline was significantly increased in mesenteric resistance arteries from hexadecanedioate-treated rats compared with controls, indicated by the shift to the left of the concentration–response curve (*P*=0.013). Relaxation to carbachol did not show any difference. Our findings indicate that hexadecanedioate is causally associated with blood pressure regulation through a novel pathway that merits further investigation.

## Abstract 2

Supplemental Digital Content is available in the text.

Hypertension is the most prevalent modifiable risk factor for cardiovascular morbidity and mortality, with the World Health Organization estimating that the current 1 billion people with high blood pressure (BP) worldwide will rise to 1.5 billion by 2020^1^. More importantly, 54% of stroke and 47% of ischemic heart disease are directly attributable to hypertension, thus primarily responsible for one quarter of the 52.8 million deaths recorded globally in 2010^2^. The causation of hypertension is multifactorial and is related to perturbations in the pathways that regulate BP through renal, renin–angiotensin–aldosterone and autonomic systems. Despite the availability of numerous drugs for the treatment of hypertension, only one third of treated patients achieve target and this can be partially explained by the complexity of BP regulatory mechanisms that are different in each individual.^3^ New drug development for hypertension has essentially stalled during the past 10 years and there is a need to discover novel biological pathways for hypertension that will help identify novel drug targets or enable effective targeting of therapy.^4^ The hypothesis generating approach using genome-wide association studies while successful at identifying common variants associated with blood pressure^5,6^ have resulted in few tractable loci and novel pathways.^7,8^ Recent advances in metabolomics enable the capture of a snap-shot of the metabolic profile of the individual, allowing for the potential identification of novel pathogenic pathways.^9–11^

In this study, we performed nontargeted metabolomic screening to discover metabolites associated with BP integrating data from dietary intake and mortality outcomes in a large female twin cohort from the United Kingdom and replicated our findings in 2 independent populations from Germany and England. Finally, we use animal models to establish causality.

## Methods

### Discovery Population

Metabolomic data were analyzed for 3980 female participants from TwinsUK^12^ without renal impairment (estimated glomerular filtration rate >60 mL/min per 1.73 m^2^) and not on any BP-lowering medications. The study was approved by St. Thomas Hospital Research Ethics Committee. All participants provided informed written consent. Further details are available in the online-only Data Supplement. TwinsUK metabolomics, and phenotypic data are publicly available on request on the department website (http://www.twinsuk.ac.uk/data-access/accessmanagement/).

### Metabolomic Profiling

Nontargeted detection and quantification of 280 structurally named biochemicals was conducted by Metabolon, Inc (Durham, NC) on 1052 serum and 5003 plasma fasting samples from TwinsUK participants as previously described.^9^

### Replication Cohorts for Hexadecanedioate

#### KORA

We included 1494 individuals comprising nearly equal numbers of males and females, with BP, metabolomics data and without renal impairment (estimated glomerular filtration rate >60 mL/min per 1.73 m^2^) from the population-based KORA S4 study (Cooperative Health Research in the Region of Augsburg).^13^

#### Hertfordshire Cohort

One thousand five hundred fifteen comprising nearly equal numbers of males and females from the Hertfordshire Cohort in whom BP and plasma hexadecanedioate levels had been measured were included in analyses. This is a unique population-based cohort of older individuals born in Hertfordshire in the 1930s and still living there now.^14^ Plasma hexadecanedioate levels were measured with mass spectrometer at the Seibersdorf Labor GmbH.

Thirty-five percent of both KORA and Hertfordshire individuals were on antihypertensive medication treatment. Therefore, 10/5 mm Hg was added to the on treatment BP in the analysis^15^ to adjust for treatment effect.

### Animal Studies

All animal procedures performed were approved by the UK Home Office. Male spontaneously hypertensive stroke-prone (SHRSP) rats^16^ and Wistar–Kyoto (WKY) rats (Harlan, Wyton, United Kingdom) were used. Eleven-week-old male WKY rats were treated with hexadecanedioic acid (250 mg/kg per day; n=6) or vehicle control (n=6) for 4 weeks. Twelve-week-old male SHRSP rats received 1% NaCl in drinking water for 3 weeks (n=6). Systolic BP (SBP) was measured by tail plethysmography in conscious, restrained animals.^17,18^ BP recordings were carried out before salt challenge or hexadecanedioic acid treatment and during the final week of treatment. An average of 6 to 8 pressure readings were taken for each rat per sitting. Statistical comparisons were made using Student *t* test. In addition, telemetry probes (TA11PAC40, Dataquest IV Data Sciences International) were implanted at 10 weeks of age with 1 week of recovery before administration of hexadecanedioic acid (250 mg/kg per day; n=5) or vehicle control (n=5) for 3 weeks in WKY rats. Statistical analysis of the radiotelemetry data was carried out using appropriate summary measures followed by Student *t* test.^19^

Mesenteric resistance artery function was assessed by wire myography. Resistance arteries were dissected from connective tissue and segments (≈2 mm in length) were mounted as ring preparations on 2 stainless steel wires in a 4-channel small vessel myograph (Danish MyoTechnology, Aarhus, Denmark). One wire was attached to a force transducer and the other to a micrometer. After a 30-minute rest period, vessels were set to normalized internal diameter (*L*_1_) to achieve optimal contraction. Internal diameter was calculated using the following equation: *L*_1_=0.0×*L*_100_ (*L*_100_ was determined using the LaPlace equation, *P*=*T*/*r*, where *P* is the effective pressure; *T*, the wall tension; and *r*, the internal radius). After further 60 minutes, contractile responses to 10 μmol/L KCl were examined, followed by washout. A cumulative concentration–response curve to noradrenaline, 10 nmol/L to 30 μmol/L was performed. In addition, vessels were preconstricted to the effective concentration (EC_50_) of noradrenaline and a concentration–response curve for carbachol (10 nmol/L–10 μmol/L) was obtained. Area under the curve data were calculated from the mesenteric artery response curves (n=10 hexadecanedioate treated and n=9 control) and statistical comparisons were made using Student *t* test.

Plasma hexadecanedioate levels were measured with mass spectrometer at the Seibersdorf Labor GmbH.

### Statistical Analysis

Statistical analysis was carried out using Stata version 11 and R. Quality control of metabolomics data was carried out as previously described.^9^ We inverse normalized the data as the metabolite concentrations were not normally distributed. To avoid spurious false-positive associations because of small sample size, we excluded metabolic traits with >20% missing values. We imputed the missing values using the minimum run day measures.

#### Metabolites Associated With SBP and Diastolic BP

Metabolites associated with SBP and diastolic BP (DBP) were identified by linear regression adjusting for age,^2^ body mass index, metabolite batch, and family relatedness. Individual metabolites significantly associated with SBP and DBP (Bonferroni *P*<8.9×10^−5^ for 280 metabolites×2 traits) were included in a backward regression model reducing the number of predictors for SBP and DBP.

As dietary factors (fruit and vegetable intake and alcohol intake) and genotype risk scores are known to affect BP to varying levels,^20^ we tested their effect on the association between the metabolites represented independent variables using random intercept linear regressions.

In KORA and Hertfordshire, the covariates in the regression model included age,^2^ sex, and body mass index.

#### Association With Mortality

Kaplan–Meier survival analysis (univariate) and Cox proportional hazards modeling (adjusted for age, body mass index, and stratified for batch effects) were used to study association of tertiles of metabolites on all-cause mortality in TwinsUK.

## Results

The overall analysis pipeline is presented in Figure S1 in the online-only Data Supplement.

### Discovery Study in TwinsUK

A total of 3080 adult females not on BP-lowering therapy were included in the analysis of 280 blood metabolites. The demographic characteristics of the study population are presented in Table 1. SBP and DBP were found to correlate with 69 and 63 metabolites, respectively (each *P*<8.9×10^−5^ after multivariate adjustment; Table S1 in the online-only Data Supplement). Backward linear regressions identified 15 metabolites independently associated with SBP or DBP—14 metabolites for SBP (*R*^2^=32%), 7 for DBP (*R*^2^=21%) with 6 in common (Table 2). Figure S2 in the online-only Data Supplement shows the bivariate correlation between the 15 identified metabolites. Adjustment for dietary and genome-wide association study risk score showed the metabolite-BP associations to be independent of these covariates (Table S2 in the online-only Data Supplement).

**Table 1.**
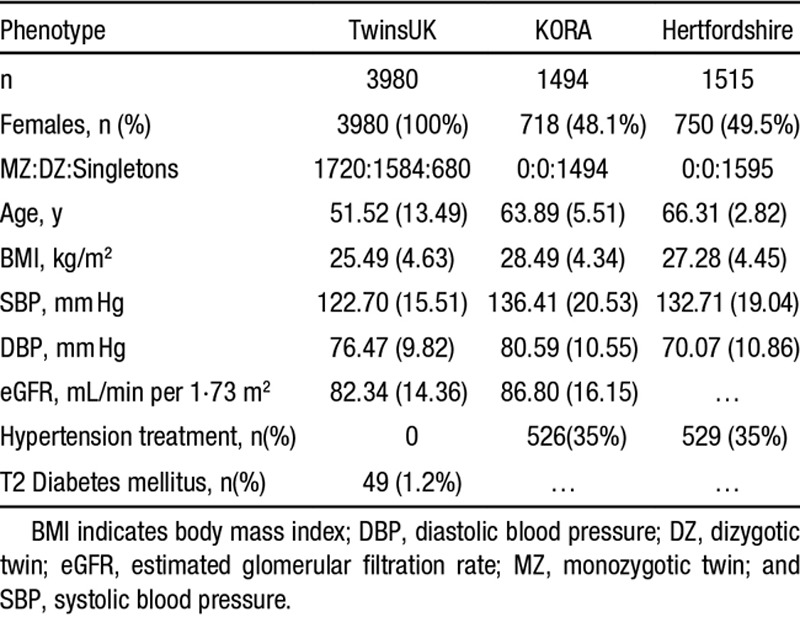
Descriptive Characteristics of the TwinsUK, KORA, and Hertfordshire Populations

**Table 2.**
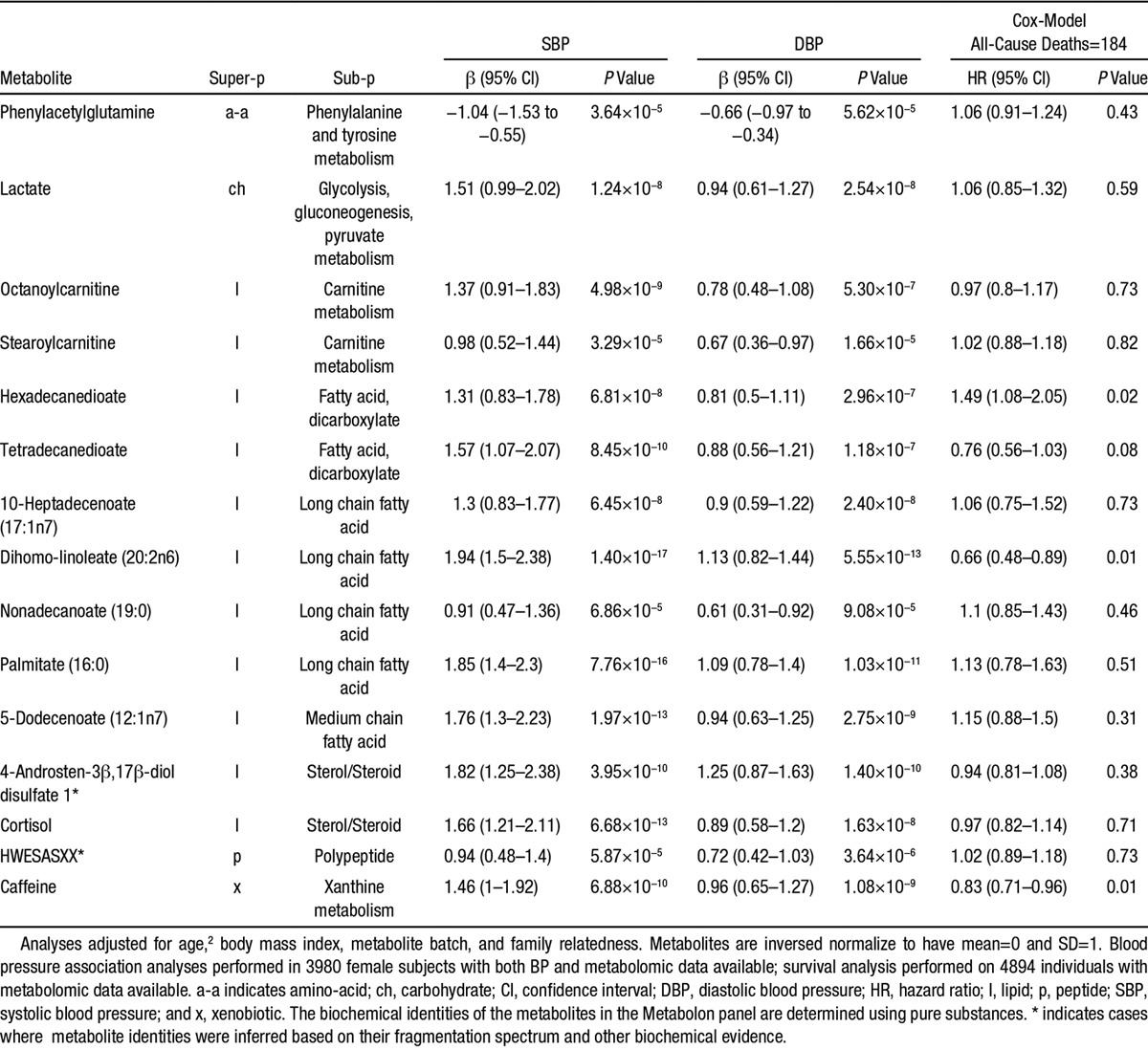
Results From Single Metabolite Analysis of the Metabolites Significant in Backward Regression and Metabolite Levels and Risk of All-Cause Mortality in TwinsUK

We then studied the association between each metabolite and all-cause mortality by performing survival analysis in the overall group of 4894 individuals in the TwinsUK cohort with available follow-up and metabolomics data. The total time at risk was 36 447 person-years with a median survival time of 6.3 years and 186 all-cause deaths. Univariate association with all-cause mortality for the 3 metabolites, such as hexadecanedioate, dihomo-linoleate (20:2n6), and caffeine are shown in the Kaplan–Meier plots (Figure S3 in the online-only Data Supplement). Multivariate-adjusted Cox proportional hazard models showed hexadecanedioate (model 1: hazard ratio, 1.49 [95% confidence interval, 1.08–2.05]; model 2: 1.71 [1.09–2.68]), dihomo-linoleate (20:2n6; model 1: 0.66 [0.48–0.89]; model 2: 0·63 [0.41–0.96]), and caffeine (model 1: 0.93 [0.72–0.97]; model 2: 0.82 [0.67–1.01]) significantly associated with all-cause mortality (Table 2; Table S3 in the online-only Data Supplement). Only hexadecanedioate showed concordant direction of effect for both BP and mortality, while in contrast, the direct association between dihomo-linoleate(20:2n6) or caffeine and blood pressure did not translate into increased mortality risk.

### Replication Analysis of top two metabolites Hexadecanedioate BP Association in KORA and Hertfordshire

The replication KORA S4 cohort included 1494 individuals (males=776 and females=718), whereas the Hertfordshire cohort included 1515 individuals (males=765 and females=750). Hexadecanedioate (SBP: β [95% confidence interval], 1.42 [0.37–2.47], *P*=0.01; DBP: 0.64 [0.09–1.19], *P*=0.02) was significantly associated with both SBP and DBP in the KORA S4 population after adjusting for covariates. Hexadecanedioate was also significantly associated with both SBP and DBP in the Hertfordshire cohort (SBP: 1.58 [0.56–2.60], *P*=0.002; DBP: 0.56 [0.02–1.1], *P*=0.04; Table S4 in the online-only Data Supplement).

### Causal Role for Hexadecanedioate in Increasing BP

**Figure 1.**
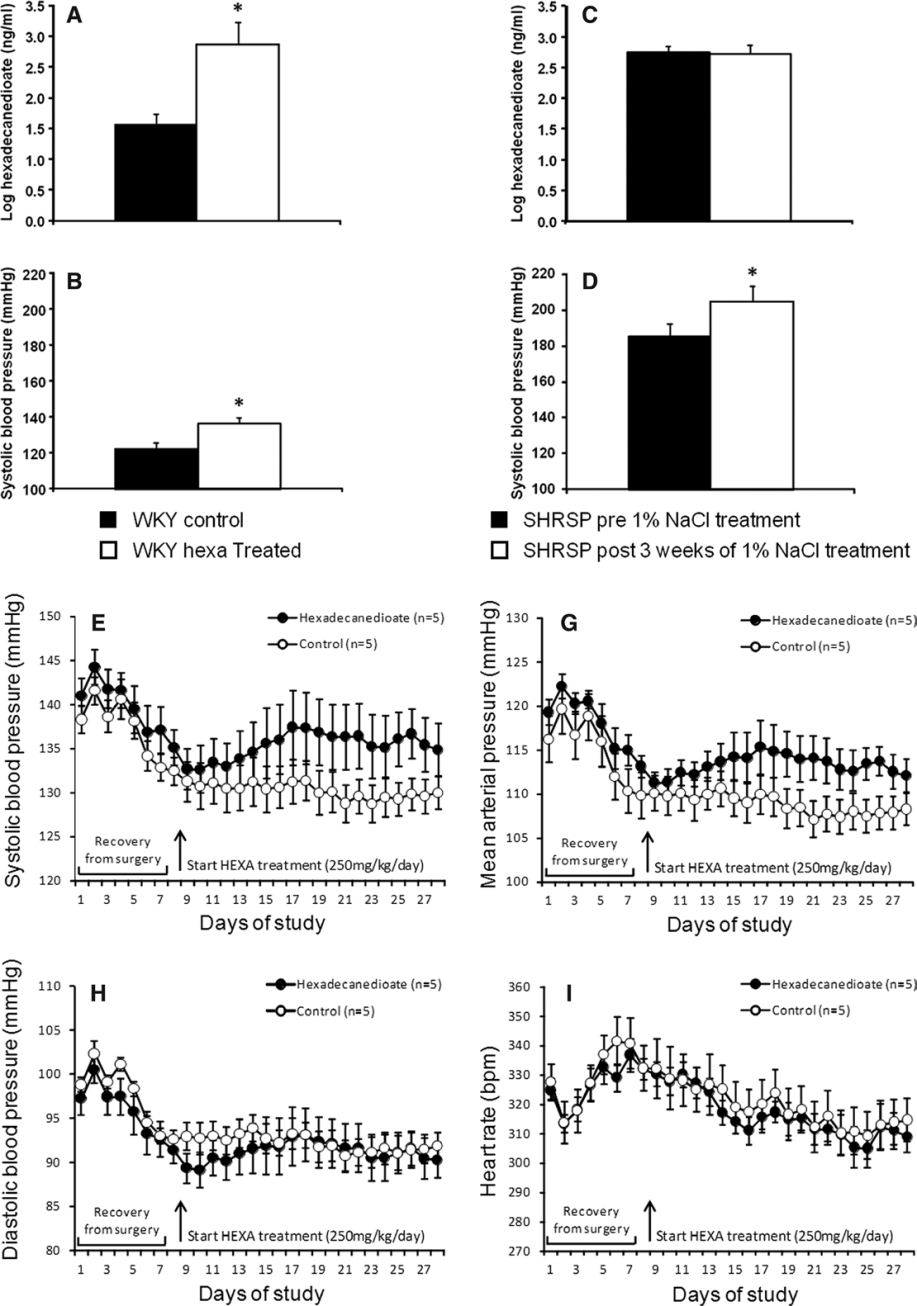
**A**, Plasma hexadecanedioate levels (ng/mL) and (**B**) systolic blood pressure (SBP; mm Hg) in Wistar–Kyoto (WKY) rats treated with 250 mg/kg per day hexadecanedioate or vehicle for 4 weeks (n=6). **C**, Plasma hexadecanedioate levels (ng/mL) and (**D**) SBP (mm Hg) in spontaneously hypertensive stroke prone (SHRSP) rats (n=6) pre- and postadministration of 1% NaCl in drinking water for 3 weeks. *P<0.05 vs respective untreated group. Radiotelemetry measurement (24-h averages) of (**E**) systolic blood pressure, (**F**) mean arterial pressure, (**G**) diastolic pressure, and (**H**) heart rate in WKY rats treated with hexadecanedioic acid (250 mg/kg per day, n=5) or vehicle (n=5) for 3 weeks.

**Figure 2.**
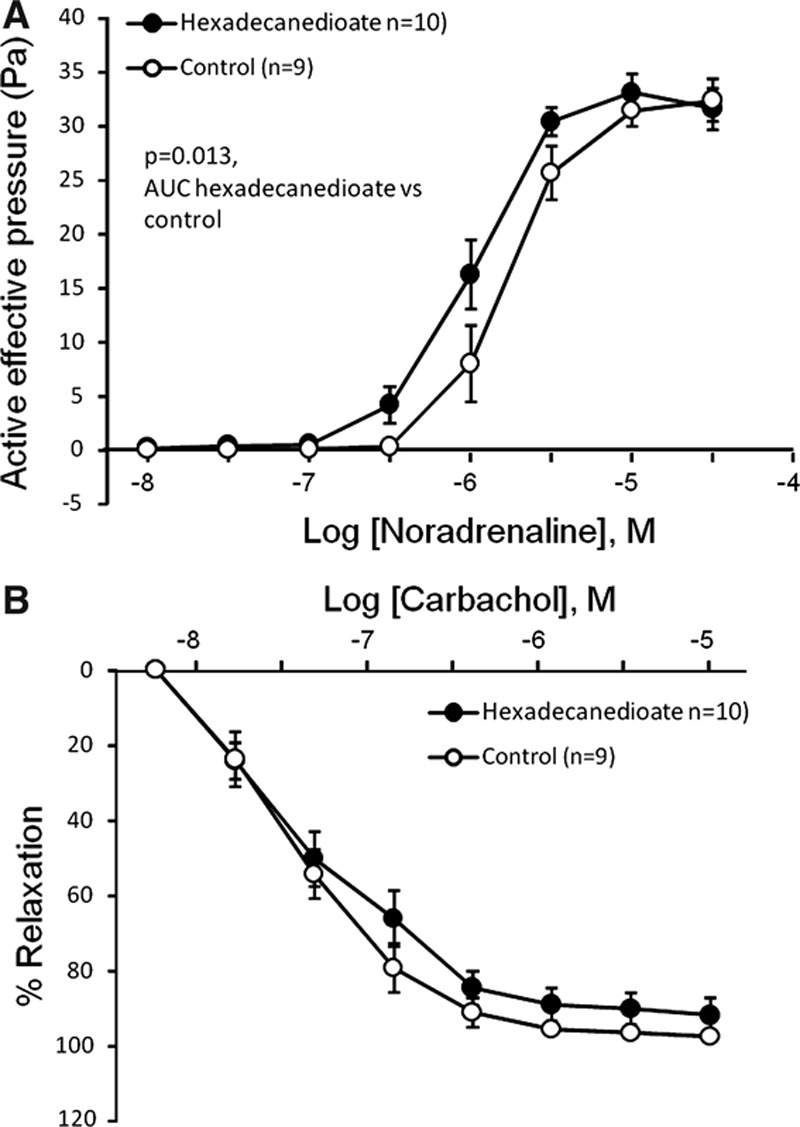
Mesenteric resistance artery contractile response to noradrenaline (**A**) and relaxation to carbachol (**B**) in control (n=9) and hexadecanedioate treated (n=10) WKY rats. AUC indicates area under the curve.

Circulating levels of hexadecanedioate were significantly increased in WKY rats after treatment with oral hexadecanedioic acid for 4 weeks compared with untreated controls (*P*=0.014; Figure 1A). Hexadecanedioate-treated WKY rats also demonstrated a small but significant increase in SBP (ΔSBP, 8.38±2.75 mm Hg; *P*=0.019) compared with untreated controls when measured by tail-cuff plethysmography (Figure 1B). The hexadecanedioic acid induced increase in BP in WKY rats was further confirmed by radiotelemetry, which demonstrated a delayed separation of SBP and mean arterial pressure between control and hexadecanedioate-treated animals occurring after day 15 of treatment, which did not attain statistical significance (*P*=0.084, SBP hexadecanedioate versus control; *P*=0.057, mean arterial pressure hexadecanedioate versus control), but did not show any separation with DBP or heart rate (Figure 1F and 1H). Three weeks of 1% NaCl administration in drinking water significantly elevated SBP in SHRSP rats (ΔSBP, 21.33±5·80 mm Hg; *P*=0.014; Figure 1D), however, plasma hexadecanedioate levels were not modified by salt challenge (Figure 1C). Baseline circulating hexadecanedioate levels were significantly higher in SHRSP rats compared with untreated age-matched WKY rats (*P*=0.0001). Vascular reactivity to noradrenaline was significantly increased in mesenteric resistance arteries from hexadecanedioate-treated rats compared with controls, indicated by the shift to the left of the concentration response curve (hexadecanedioate area under the curve 100.8±9.3 versus control area under the curve 81.7±8.8; *P*=0.013; Figure 2A). Relaxation to carbachol was not different in mesenteric resistance arteries from control and hexadecanedioate-treated WKY rats (Figure 2B).

## Discussion

In the largest investigation to date on metabolomic profiling for BP, we have identified a putative novel pathway for BP regulation involving a dicarboxylic acid (hexadecanedioate) with a causal role supported by in vivo studies in rats. After screening 280 metabolites in the discovery TwinsUK cohort, we identified 77 metabolites significantly associated with SBP or DBP. To limit the number of metabolites to take forward for replication, we narrowed the list down to 15 independent metabolites through a series of sequential analytic steps to address the observed correlations between metabolite levels. These 15 metabolite signals were also independent of fruit/vegetable or alcohol intake and BP genome-wide association study risk score all of which affect BP to varying levels.^20^ To minimize the risk of reverse causation as an explanation for our findings, we tested the association between these metabolites with all-cause mortality in the discovery cohort. Although incident hypertension or cardiovascular mortality would have been ideal longitudinal phenotypes to analyze, these were unavailable in the discovery cohort. As high BP does increase the risk of both all-cause and cardiovascular mortality, we used all-cause mortality to filter metabolites for replication.

Only 3 metabolites (dihomo-linoleate (20:2n6), hexadecanedioate, and caffeine) showed significant associations with mortality in TwinsUK. More importantly, only 1 metabolite (hexadecanedioate) of the 3 showed directionally consistent and significant associations with both BP and mortality suggesting a possible sustained detrimental effect of higher levels of the metabolite through increased BP. This is in contrast to the other 2 metabolites, dihomo-linoleate (20:2n6) and caffeine, which did not increase mortality, despite showing positive associations with BP. In addition, we performed survival analysis on other dicarboxylic acids (tetradecanedioate, octadecanedioate, and dodecanedioate) and none of them showed significant concordant association with mortality. Although our discovery analysis included only women who were not on any BP-lowering medications, we are able to demonstrate generalizability of our results for hexadecanedioate through replication in the KORA and Hertfordshire study, both of which included male and female subjects and hypertensive subjects on treatment. Our discovery results are maintained even after inclusion of individuals from the TwinsUK cohort who were on BP-lowering medications (Table S5 in the online-only Data Supplement). We recognize that the lack of concordant mortality effect with BP association for dihomo-linoleate (20:2n6) and caffeine does not conclusively exclude a pathological effect, but we think further analyses of dihomo-linoleate (20:2n6) in larger cohorts with more events is warranted before further functional studies are conducted. The lack of a detrimental effect on survival for caffeine levels reflects the acute effect of caffeine on blood pressure, which varies with intake. We had a priori excluded individuals with renal impairment in our analysis and adjusting for estimated glomerular filtration rate did not alter the BP hexadecanedioate association. There is evidence that long-chain fatty acids, such as docosahexaenoic acid influence BP potentially through an effect on large-conductance Ca(2+)- and voltage-activated K+ (BK) channels,^21^ but this was not evident in our study.

We performed 3 in vivo experiments to establish causality of hexadecanedioate on BP regulation. In the first, after oral intake of hexadecanedioate, normotensive WKY rats showed an increase in circulating hexadecanedioate levels along with an increase in BP, both of which were statistically significant. In the second experiment, we measured hexadecanedioate levels in the SHRSP rat before and after administration of salt, which resulted in a further increase in BP but no change in circulating hexadecanedioate levels. Interestingly hexadecanedioate levels in the SHRSP rats were higher than that of WKY rats at baseline, and levels were similar between SHRSP and posthexadecanedioate WKY rats. We used radio-telemetry to provide confirmation of our tail-cuff plethysmography data and to determine the time course of the hexadecanedioate-induced rise in blood pressure. From the radio-telemetry data, the increase in SBP and mean arterial pressure is not immediate after hexadecanedioate administration, with separation of SBP and mean arterial pressure between control and hexadecanedioate-treated animals occurring after day 15 of treatment. This suggests that the mechanism by which hexadecanedioate affects BP is probably through secondary downstream pathways activated by higher hexadecanedioate levels. The increased vascular reactivity to noradrenaline shown by mesenteric arteries of hexadecanedioate-treated rats point to a vascular mechanism underlying the association of hexadecanedioate and blood pressure. Although these are preliminary data, overall they provide strong corroborative evidence of a causal role for hexadecanedioate in modulating blood pressure and support further research in validating and elucidating the mechanisms.

Hexadecanedioic acid is a long-chain dicarboxylic acid, which is generated during fatty acid ω-oxidation and thence metabolized by β-oxidation in peroxisomes. ω-oxidation is a minor metabolic pathway that occurs in the endoplasmic reticulum and also contributes to 5% to 10% of total fatty acid metabolism in the liver. ω-Oxidation is increased in conditions that are characterized by increased levels of mono-carboxylic free fatty acid (obesity, starvation, diabetes mellitus, and chronic alcohol consumption), as well as disturbances in β-oxidation.^22,23^ A lack of carnitine can lead to increased ω-oxidation.^24^ In our data, we find that levels of hexadecanedioate are indeed negatively correlated with carnitine (β=−0.05; 95% confidence interval, [−0.08 to −0.01]; *P*=0.01].

Multiple strands of evidence from related pathways point to potential mechanisms that can inform future studies to dissect the hexadecanedioate BP association. Nonesterified fatty acids reduce Na^+^K^+^-ATPase activity in vascular smooth muscle cells through increase in intracellular Na^+^ and decrease of passive Na^+^/Ca^2+^ exchange or through partial depolarization of cell membrane and activation of voltage-dependent Ca^2+^ channels resulting in increased intracellular Ca^2+^ concentration and a relative elevation of vascular tone, thus promoting the development of hypertension.^25,26^

Interestingly, a recent report showed multiple dicarboxylic acids, including hexadecanedioate, to be significantly accumulated in pulmonary arterial hypertension tissues indicating a disruption of β-oxidation and an increase of ω-oxidation in this condition and pointing to a putative role in elevating pressure in both the systemic and the pulmonary circulations.^27^ Indeed metabolic dysfunction and acquired mitochondrial abnormalities leading to abnormal glycolytic and fatty acid metabolism are now recognized as a potential biological mechanism leading to both pulmonary vascular remodeling with aberrant cellular proliferation and apoptosis, and in the development of right ventricular failure.^28,29^ Fatty acid metabolism could offer a novel therapeutic pathway, which would potentially target both pulmonary vasculature and the right ventricle.

Some of the specific functional effects of hexadecanedioate have been studied using β,β′-tetramethylhexadecanedioic acid (MEDICA 16), which is not metabolized and hence the effects of downstream metabolic products that may mask the effects of hexadecanedioate are minimized. MEDICA 16 has been shown to be effective as a hypolipidemic and antiobesity/anti-insulin resistance agent in experimental models,^30,31^ and has a liver-specific calorigenic-thyromimetic action characterized by a decrease in liver phosphate potential and liver redox potential with an increase in oxygen consumption, but its effect on BP has not been studied thus far.^32,33^ Endogenous hexadecanedioate may be a putative marker either for the perturbations in immunologic processes or lipid β-oxidation that lead to high blood pressure, and further studies are needed to clarify the mechanism. Human metabolomic studies are potentially confounded by diet or drug effects, as well as other comorbidities.^34^ In our discovery study, we tested for the effect of diet and alcohol intake, excluded individuals who were on antihypertensive therapy and looked for corroborative evidence of increased mortality.

We note some limitations to our study. All the human metabolomics analysis is cross-sectional so we cannot dissect causality from it alone. We used all-cause mortality rather than incident hypertension or cardiovascular mortality because of lack of data. Our 2-stage approach was designed to take forward a limited number of metabolites for replication to attenuate the multiple testing burden and the high false-positive rates seen in high-throughput discovery studies. We recognize that hexadecanedioate may be a low-hanging fruit and that there will exist other valid metabolites that are associated with BP. Although our in vivo studies support a role for hexadecanedioate in BP regulation, the exact causal pathway is not established. The increased vascular reactivity to noradrenaline in hexadecanedioate-treated rats may be either a consequence of hypertension or a cause, and this needs to be elucidated in future experiments.

### Perspectives

Using a multilayered approach comprising metabolomic profiling in twins followed by replication studies and in vivo experiments, we have uncovered a putative novel pathway of BP regulation involving a dicarboxylic acid, hexadecanedioate. Preliminary studies from pulmonary hypertension suggesting an accumulation of dicarboxylic acids in this condition supports a possible vascular role for dicarboxylic acids in increasing pressure in both the systemic and pulmonary circulation. Studies on fatty acid metabolism could offer a novel therapeutic pathway, which would potentially target both pulmonary and systemic hypertension. Our finding may indeed be a low-hanging fruit but as it points to a previously unknown pathway for blood pressure regulation these results should stimulate further studies specifically along 2 strands—(1) confirm and elucidate the mechanistic underpinnings of the role of fatty acid ω-oxidation in blood pressure regulation and (2) identify other true causal metabolite associations.

## Acknowledgments

We wish to express our appreciation to all study participants of the TwinsUK, KORA, and Hertfordshire studies.

## Sources of Funding

This work was supported by: the EU Framework Programme 7 small-scale focused research collaborative project EurHEALTHAging 277849; Metabolomic analysis was funded by Pfizer; TwinsUK was funded by the Wellcome Trust (Ref: 081878/Z/06/Z); European Community’s Seventh Framework Programme (FP7/2007–2013). The study also receives support from the National Institute for Health Research (NIHR) Clinical Research Facility at Guy’s and St. Thomas’ National Health Service (NHS) Foundation Trust and NIHR Biomedical Research Centre based at Guy’s and St Thomas’ NHS Foundation Trust and King’s College London. T. Spector is an NIHR senior Investigator and is holder of an ERC Advanced Principal Investigator award. S.-Y. Shin is supported by a Post-Doctoral Research Fellowship from the Oak Foundation. N. Soranzo team is supported by the Wellcome Trust (Grant Codes WT098051 and WT091310), the EUFP7 (EPIGENESYS Grant Code 257082 and BLUEPRINT Grant Code HEALTH-F5-2011–282510). The KORA research platform (KORA, Cooperative Research in the Region of Ausburg) was initiated and financed by the Helmholtz Zentrum München—German Research Center for Envirnomental Health, which is funded by the German Federal Ministry of Education and Research and by the State of Bavaria. Furthermore, KORA research was supported within the Munich Center of Health Sciences (MC Health), Ludwing-Maximilians-Universität, as part of LMUinnovativ. The Hertfordshire Cohort Study was supported by the Medical Research Council (MRC) of Great Britain; Arthritis Research UK; and the International Osteoporosis Foundation. The work herein was also supported by the NIHR Nutrition Biomedical Research Centre, University of Southampton, and the NIHR Musculoskeletal Biomedical Research Unit, University of Oxford.

## Disclosures

R.P. Mohney is an employee of Metabolon, Inc. M.J. Brosnan is full time employee and share holder of Pfizer. J. Trimmer is a share holder of Pfizer. The other authors report no conflicts.

## Supplementary Material

## Novelty and Significance

### What Is New?

- From a pool of 77 metabolites, which showed significant associations with systolic blood pressure (BP) or diastolic BP, we narrowed the list down to three metabolites (dihomo-linoleate (20:2n6), hexadecanedioate, and caffeine) that were also significantly associated with mortality in the same cohort and replicated in the KORA and Hertfordshire populations.
- Only hexadecanedioate showed concordant directionality of effect for both BP and mortality.
- We showed that hexadecanedioate is causally related to BP change using an animal model.

### What Is Relevant?

- The results presented contribute to advance our understanding of the causation of hypertension by finding a new potential pathway of BP regulation.

### Summary

Circulating levels of the dicarboxylic acid hexadecanedioate points to a novel pathway for BP regulation that merits further study.
