# Supplementary material for: Metabolomic Identification of a Novel Pathway of Blood Pressure Regulation Involving Hexadecanedioate
Source: Hypertension. 2015 Jul 8;66(2):422–9. doi: 10.1161/HYPERTENSIONAHA.115.05544 (PMC4490909; doi:10.1161/HYPERTENSIONAHA.115.05544)

**Online Supplement**

**Metabolomic identification of a novel pathway of blood pressure regulation involving hexadecanedioate**

Cristina Menni^1,*^ , Delyth Graham^2,*^, Gabi Kastenmüller^3^, Nora HJ Alharbi^2^, Safaa Md Alsanosi^2^, Martin McBride^2^, Massimo Mangino^1^ , Philip Titcombe^4^, So-Youn Shin^5,6^, Maria Psatha^1^ , Thomas Geisendorfer^7^, Anja Huber^7^, Annette Peters^8,9^, Rui Wang-Sattler^9^, Tao Xu^9^, Mary Julia Brosnan^10^ , Jeff Trimmer^11^, Christian Reichel^7^, Robert P Mohney^12^, Nicole Soranzo^5^, Mark H Edwards^4^, Cyrus Cooper^4,13^, Alistair C Church^14^, Karsten Suhre^3,15^ , Christian Gieger^8^, Anna F Dominiczak^2^, Tim D Spector^1^ , Sandosh Padmanabhan^2,17^, Ana M Valdes^1,16, 17^.

^1^ Department of Twin Research & Genetic Epidemiology, King's College London, London, UK; ^2^ Institute of Cardiovascular and Medical Sciences, College of Medical, Veterinary and Life Sciences, University of Glasgow, Glasgow, UK; ^3^ Institute of Bioinformatics and Systems Biology, Helmholtz Zentrum München, Germany; ^4^ Faculty of Medicine, University of Southampton, UK; ^5^ Wellcome Trust Sanger Institute Human Genetics, Hinxton, UK; ^6^ MRC Integrative Epidemiology Unit, School of Social and Community Medicine, University of Bristol, Bristol, UK; ^7^Chemical Analytics, Seibersdorf Labor GmbH, Seibersdorf, Austria; ^8^ Institute of Epidemiology II, Helmholtz Zentrum München, Germany; ^9^ Research Unit Molecular Epidemiology, Helmholtz Zentrum München, Germany; ^10^ Cardiovascular and Metabolic Diseases, Pfizer Worldwide Research and Development, Cambridge, MA, USA; ^11^ Edison Pharmaceuticals, Mountain View, USA; ^12^ Metabolon, Inc., Durham, USA; ^13^ Nuffield Department of Orthopaedics, Rheumatology and Musculoskeletal Sciences, University of Oxford, UK; ^14^ Scottish Pulmonary Vascular Unit, Golden jubilee Hospital, Glasgow, UK; ^15^ Department of Physiology and Biophysics, Weill Cornell Medical College in Qatar, Education City, Qatar Foundation, Doha, Qatar; ^16^ Academic Rheumatology, University of Nottingham, Nottingham, UK; ^17^ These authors contributed equally to this work; * These authors contributed equally to this work.

**Short title**: hexadecanedioate and blood pressure.

**Corresponding author:**

Prof Sandosh Padmanabhan

Institute of Cardiovascular and Medical Sciences; College of Medical, Veterinary and Life Sciences; University of Glasgow; 126 University Place; Glasgow G12 8TA.

Telephone: +44 (0) 1413302228; Fax: +44 (0)141 330 6997; Email: sandosh.padmanabhan@glasgow.ac.uk

# Methods

## A. TwinsUK – phenotype assessment and modelling.

*Blood pressure measurement*

Clinic BP was measured by a trained nurse using either the Marshall mb02, the Omron Mx3 or the Omron HEM713C Digital Blood Pressure Monitors performed with the patient in the sitting position for at least 3 minutes. At each visit, the cuff was placed on the subject's arm so that it was approximately 2-3 cm above the elbow joint of the inner arm, with the air tube lying over the brachial artery. The subject’s arm was placed on the table or supported with the palm facing upwards, so that the tab of the cuff was placed at the same level of the heart. Three measurements were taken with an interval of approximately 1 minute between each reading. The second and third measures were subsequently recorded. BP measures were normally distributed.

*Dietary and other data*

Each subject completed a 131-item Food Frequency Questionnaire (FFQ) which was developed for the EPIC (European Prospective Investigation into Cancer and Nutrition) Norfolk study[^1^](#_ENREF_1). Macro and micro nutrient intakes were calculated from an established nutrient database[^2^](#_ENREF_2). For each food group, the frequency of intake (serving/wk) was adjusted for the total energy intake using the residual method. The energy-adjusted intakes were standardized and used in the principal components analysis (PCA) as previously described[^3^](#_ENREF_3). The dietary analysis was performed on five principal components which account for 22% of the total variance: fruit and vegetable, high alcohol, traditional English diet, hypo-caloric dieting and low meat.

TwinsUK data are publicly available upon request on the department website (<http://www.twinsuk.ac.uk/data-access/accessmanagement/>).

# References

1. Bingham SA, Welch AA, McTaggart A, Mulligan AA, Runswick SA, Luben R, Oakes S, Khaw KT, Wareham N, Day NE. Nutritional methods in the european prospective investigation of cancer in norfolk. *Public health nutrition*. 2001;4:847-858.

2. Holland B, Welch AA, Unwin ID, Buss DH, Paul AA, Southgate DAT. *The composition of foods*. London: HMSO Ministry of Agriculture, Fisheries and Food and Royal Chemistry; 1991.

3. Menni C, Zhai G, Macgregor A, Prehn C, Romisch-Margl W, Suhre K, Adamski J, Cassidy A, Illig T, Spector TD, Valdes AM. Targeted metabolomics profiles are strongly correlated with nutritional patterns in women. *Metabolomics*. 2013;9:506-514.

##

## Table S1. List of significant metabolites in one or more comparison in the TwinsUK cohort. All analyses are adjusted for age, age^2^, BMI, batch effect and family relatedness.

|  |  |  | **SBP** | | **DBP** | |
| --- | --- | --- | --- | --- | --- | --- |
| **Metabolite** | **Super-p** | **Sub-p** | **Beta[95%CI]** | **P** | **Beta[95%CI]** | **P** |
| alanine | a-a | Alanine and aspartate metabolism | 1.09[0.58;1.59] | 2.85E-05 | 0.46[0.14;0.78] | 4.65E-03 |
| 2-hydroxybutyrate (AHB) | a-a | Cysteine, methionine, SAM, taurine metabolism | 1.67[1.16;2.17] | 1.01E-10 | 1.09[0.76;1.42] | 9.41E-11 |
| cysteine | a-a | Cysteine, methionine, SAM, taurine metabolism | 1[0.49;1.52] | 1.49E-04 | 0.69[0.36;1.03] | 4.20E-05 |
| 3-(4-hydroxyphenyl)lactate | a-a | Phenylalanine & tyrosine metabolism | 1.04[0.54;1.54] | 5.19E-05 | 0.78[0.45;1.12] | 4.63E-06 |
| p-cresol sulfate | a-a | Phenylalanine & tyrosine metabolism | -1.03[-1.5;-0.56] | 1.62E-05 | -0.71[-1.01;-0.4] | 6.51E-06 |
| phenylacetylglutamine | a-a | Phenylalanine & tyrosine metabolism | -1.04[-1.53;-0.55] | 3.64E-05 | -0.66[-0.97;-0.34] | 5.62E-05 |
| phenyllactate (PLA) | a-a | Phenylalanine & tyrosine metabolism | 0.9[0.44;1.36] | 1.44E-04 | 0.69[0.38;1] | 1.59E-05 |
| 3-indoxyl sulfate | a-a | Tryptophan metabolism | -1.24[-1.71;-0.78] | 1.98E-07 | -0.55[-0.86;-0.24] | 4.47E-04 |
| dimethylarginine (SDMA + ADMA) | a-a | Urea cycle; arginine-, proline-, metabolism | -1.26[-1.76;-0.77] | 6.70E-07 | -0.65[-0.97;-0.32] | 9.44E-05 |
| 3-methyl-2-oxobutyrate | a-a | Valine, leucine and isoleucine metabolism | 0.84[0.38;1.3] | 3.30E-04 | 0.67[0.36;0.99] | 2.95E-05 |
| 3-methyl-2-oxovalerate | a-a | Valine, leucine and isoleucine metabolism | 1[0.54;1.46] | 1.93E-05 | 0.57[0.26;0.89] | 3.71E-04 |
| alpha-hydroxyisovalerate | a-a | Valine, leucine and isoleucine metabolism | 1.31[0.83;1.78] | 7.63E-08 | 0.93[0.61;1.24] | 1.04E-08 |
| levulinate (4-oxovalerate) | a-a | Valine, leucine and isoleucine metabolism | 1.13[0.66;1.61] | 2.85E-06 | 0.44[0.13;0.75] | 5.98E-03 |
| erythronate* | ch | Aminosugars metabolism | 1.2[0.65;1.75] | 1.79E-05 | 0.65[0.31;1] | 2.25E-04 |
| mannose | ch | Fructose, mannose, galactose, starch, and sucrose metabolism | 1.09[0.58;1.6] | 2.81E-05 | 0.39[0.06;0.72] | 2.10E-02 |
| glucose | ch | Glycolysis, gluconeogenesis, pyruvate metabolism | 1.34[0.8;1.87] | 8.96E-07 | 0.68[0.34;1.02] | 7.93E-05 |
| lactate | ch | Glycolysis, gluconeogenesis, pyruvate metabolism | 1.51[0.99;2.02] | 1.24E-08 | 0.94[0.61;1.27] | 2.54E-08 |
| bilirubin (E,E)* | c&v | Hemoglobin and porphyrin metabolism | 0.91[0.44;1.38] | 1.55E-04 | 0.66[0.35;0.97] | 3.27E-05 |
| alpha-ketoglutarate | e | Krebs cycle | 0.76[0.26;1.27] | 3.04E-03 | 0.7[0.38;1.01] | 1.62E-05 |
| citrate | e | Krebs cycle | 1.11[0.57;1.66] | 6.85E-05 | 0.71[0.37;1.05] | 5.58E-05 |
| malate | e | Krebs cycle | 1.58[1.07;2.09] | 1.27E-09 | 0.99[0.66;1.32] | 6.28E-09 |
| acetylphosphate | e | Oxidative phosphorylation | 1.07[0.53;1.61] | 1.04E-04 | 0.73[0.39;1.07] | 2.54E-05 |
| 2-tetradecenoyl carnitine | l | Carnitine metabolism | 0.93[0.47;1.39] | 6.97E-05 | 0.73[0.43;1.04] | 1.94E-06 |
| acetylcarnitine | l | Carnitine metabolism | 1.4[0.93;1.87] | 5.79E-09 | 0.98[0.67;1.29] | 6.60E-10 |
| decanoylcarnitine | l | Carnitine metabolism | 1.13[0.69;1.57] | 6.18E-07 | 0.65[0.36;0.95] | 1.67E-05 |
| hexanoylcarnitine | l | Carnitine metabolism | 1.48[1.01;1.96] | 1.27E-09 | 0.85[0.53;1.16] | 2.05E-07 |
| octanoylcarnitine | l | Carnitine metabolism | 1.37[0.91;1.83] | 4.98E-09 | 0.78[0.48;1.08] | 5.30E-07 |
| oleoylcarnitine | l | Carnitine metabolism | 1.06[0.6;1.53] | 7.69E-06 | 0.69[0.39;1] | 9.96E-06 |
| palmitoylcarnitine | l | Carnitine metabolism | 1.45[0.98;1.91] | 1.72E-09 | 0.98[0.67;1.28] | 6.25E-10 |
| stearoylcarnitine | l | Carnitine metabolism | 0.98[0.52;1.44] | 3.29E-05 | 0.67[0.36;0.97] | 1.66E-05 |
| dihomo-linolenate (20:3n3 or n6) | l | Essential fatty acid | 1.35[0.9;1.8] | 4.45E-09 | 0.73[0.42;1.04] | 3.19E-06 |
| docosapentaenoate (n3 DPA; 22:5n3) | l | Essential fatty acid | 1.5[1.03;1.97] | 4.46E-10 | 0.99[0.67;1.31] | 1.13E-09 |
| linoleate (18:2n6) | l | Essential fatty acid | 1.61[1.15;2.06] | 4.15E-12 | 0.87[0.56;1.18] | 3.17E-08 |
| linolenate [alpha or gamma; (18:3n3 or 6)] | l | Essential fatty acid | 1.47[1.03;1.91] | 5.88E-11 | 0.89[0.59;1.19] | 4.99E-09 |
| dodecanedioate | l | Fatty acid, dicarboxylate | 1.47[0.96;1.99] | 2.04E-08 | 0.81[0.47;1.14] | 2.30E-06 |
| hexadecanedioate | l | Fatty acid, dicarboxylate | 1.31[0.83;1.78] | 6.81E-08 | 0.81[0.5;1.11] | 2.96E-07 |
| octadecanedioate | l | Fatty acid, dicarboxylate | 1.17[0.7;1.63] | 8.90E-07 | 0.65[0.35;0.95] | 2.29E-05 |
| tetradecanedioate | l | Fatty acid, dicarboxylate | 1.57[1.07;2.07] | 8.45E-10 | 0.88[0.56;1.21] | 1.18E-07 |
| glycerol | l | Glycerol metabolism | 2.04[1.5;2.58] | 1.90E-13 | 1.17[0.83;1.51] | 1.98E-11 |
| myo-inositol | l | Inositol metabolism | 1.11[0.59;1.63] | 3.00E-05 | 0.75[0.42;1.08] | 8.60E-06 |
| 3-hydroxybutyrate (BHBA) | l | Ketone bodies | 1.01[0.54;1.48] | 2.39E-05 | 0.7[0.4;1] | 4.78E-06 |
| 10-heptadecenoate (17:1n7) | l | Long chain fatty acid | 1.3[0.83;1.77] | 6.45E-08 | 0.9[0.59;1.22] | 2.40E-08 |
| 10-nonadecenoate (19:1n9) | l | Long chain fatty acid | 1.34[0.89;1.8] | 6.76E-09 | 0.97[0.66;1.28] | 9.65E-10 |
| dihomo-linoleate (20:2n6) | l | Long chain fatty acid | 1.94[1.5;2.38] | 1.40E-17 | 1.13[0.82;1.44] | 5.55E-13 |
| eicosenoate (20:1n9 or 11) | l | Long chain fatty acid | 1.45[0.99;1.91] | 8.03E-10 | 0.94[0.63;1.24] | 2.54E-09 |
| margarate (17:0) | l | Long chain fatty acid | 1.42[0.95;1.89] | 2.85E-09 | 0.87[0.56;1.19] | 6.92E-08 |
| myristate (14:0) | l | Long chain fatty acid | 1.77[1.3;2.24] | 1.99E-13 | 1.05[0.73;1.36] | 1.79E-10 |
| myristoleate (14:1n5) | l | Long chain fatty acid | 1.46[1;1.93] | 7.06E-10 | 0.86[0.54;1.18] | 1.40E-07 |
| nonadecanoate (19:0) | l | Long chain fatty acid | 0.91[0.47;1.36] | 6.86E-05 | 0.61[0.31;0.92] | 9.08E-05 |
| oleate (18:1n9) | l | Long chain fatty acid | 1.43[0.99;1.87] | 2.81E-10 | 0.88[0.57;1.19] | 1.99E-08 |
| palmitate (16:0) | l | Long chain fatty acid | 1.85[1.4;2.3] | 7.76E-16 | 1.09[0.78;1.4] | 1.03E-11 |
| palmitoleate (16:1n7) | l | Long chain fatty acid | 1.66[1.19;2.12] | 2.60E-12 | 0.99[0.68;1.31] | 8.70E-10 |
| pentadecanoate (15:0) | l | Long chain fatty acid | 0.91[0.41;1.42] | 4.10E-04 | 0.66[0.34;0.98] | 6.44E-05 |
| stearate (18:0) | l | Long chain fatty acid | 1.51[1.07;1.95] | 2.83E-11 | 0.94[0.63;1.25] | 2.20E-09 |
| stearidonate (18:4n3) | l | Long chain fatty acid | 1.04[0.59;1.5] | 6.18E-06 | 0.66[0.35;0.97] | 2.86E-05 |
| 1-arachidonoylglycerophosphoethanolamine* | l | Lysol | 1.01[0.57;1.46] | 9.28E-06 | 0.43[0.13;0.73] | 5.14E-03 |
| 1-myristoylglycerophosphocholine | l | Lysol | 1.05[0.6;1.5] | 4.40E-06 | 0.58[0.29;0.88] | 1.12E-04 |
| 1-palmitoleoylglycerophosphocholine* | l | Lysol | 0.97[0.51;1.44] | 4.68E-05 | 0.54[0.23;0.85] | 7.36E-04 |
| 10-undecenoate (11:1n1) | l | Medium chain fatty acid | 0.96[0.51;1.41] | 3.27E-05 | 0.62[0.33;0.92] | 3.46E-05 |
| 5-dodecenoate (12:1n7) | l | Medium chain fatty acid | 1.76[1.3;2.23] | 1.97E-13 | 0.94[0.63;1.25] | 2.75E-09 |
| caprate (10:0) | l | Medium chain fatty acid | 1.41[0.95;1.87] | 2.20E-09 | 0.6[0.3;0.9] | 9.20E-05 |
| laurate (12:0) | l | Medium chain fatty acid | 1.52[1.04;2] | 6.91E-10 | 0.68[0.36;1] | 3.66E-05 |
| 1-palmitoylglycerol (1-monopalmitin) | l | Monoacylglycerol | 1.58[1.09;2.07] | 2.97E-10 | 0.79[0.48;1.1] | 5.60E-07 |
| 1-stearoylglycerol (1-monostearin) | l | Monoacylglycerol | 1.31[0.82;1.79] | 1.36E-07 | 0.62[0.31;0.93] | 7.81E-05 |
| 4-androsten-3beta,17beta-diol disulfate 1* | l | Sterol/Steroid | 1.82[1.25;2.38] | 3.95E-10 | 1.25[0.87;1.63] | 1.40E-10 |
| cholesterol | l | Sterol/Steroid | 1.41[0.85;1.98] | 9.03E-07 | 0.93[0.57;1.28] | 3.49E-07 |
| cortisol | l | Sterol/Steroid | 1.66[1.21;2.11] | 6.68E-13 | 0.89[0.58;1.2] | 1.63E-08 |
| cortisone | l | Sterol/Steroid | 1.35[0.88;1.81] | 1.35E-08 | 0.86[0.55;1.16] | 3.75E-08 |
| urate | n | Purine metabolism, urate metabolism | 1.09[0.57;1.6] | 3.36E-05 | 0.68[0.34;1.02] | 8.43E-05 |
| gamma-glutamylglutamine | p | gamma-glutamyl | -1.01[-1.49;-0.53] | 3.87E-05 | -0.6[-0.91;-0.29] | 1.68E-04 |
| HWESASXX* | p | Polypeptide | 0.94[0.48;1.4] | 5.87E-05 | 0.72[0.42;1.03] | 3.64E-06 |
| erythritol | x | Sugar, sugar substitute, starch | 1.19[0.63;1.75] | 2.97E-05 | 0.74[0.38;1.09] | 4.23E-05 |
| 1,7-dimethylurate | x | Xanthine metabolism | 0.95[0.46;1.43] | 1.34E-04 | 0.67[0.35;0.98] | 3.95E-05 |
| caffeine | x | Xanthine metabolism | 1.46[1;1.92] | 6.88E-10 | 0.96[0.65;1.27] | 1.08E-09 |
| paraxanthine | x | Xanthine metabolism | 1.2[0.74;1.66] | 3.63E-07 | 0.76[0.47;1.06] | 5.14E-07 |
| theobromine | x | Xanthine metabolism | 0.91[0.45;1.36] | 8.87E-05 | 0.42[0.12;0.71] | 5.54E-03 |
| theophylline | x | Xanthine metabolism | 1.3[0.86;1.75] | 1.14E-08 | 0.83[0.54;1.13] | 3.84E-08 |

a-a = amino acid, ch = carbohydrate, c&v=cofactor and vitamins, e=energy, l= lipid, n-=nucleotide, p=peptide, x=xenobiotic.

## Table S2. Multivariate analysis between metabolites and SBP, DBP after adjustment for dietary variables and genotype score.

|  | **SBP** | | | | **DBP** | | | |
| --- | --- | --- | --- | --- | --- | --- | --- | --- |
|  | **adjusting for dietary variables** | | **adjusting for dietary variables and genotype score** | | **adjusting for dietary variables** | | **adjusting for dietary variables and genotype score** | |
| **Metabolite** | **Beta[95%CI]** | **P** | **Beta[95%CI]** | **P** | **Beta[95%CI]** | **P** | **Beta[95%CI]** | **P** |
| phenylacetylglutamine | -1.1[-1.68;-0.53] | 1.88x10^-4^ | -1.02[-1.76;-0.29] | 6.32x10^-3^ | -0.6[-0.96;-0.24] | 1.20x10^-3^ | -0.54[-0.98;-0.1] | 1.75x10^-2^ |
| lactate | 1.51[0.92;2.1] | 5.38x10^-7^ | 1.72[0.96;2.48] | 1.07x10^-5^ | 0.82[0.45;1.19] | 1.72x10^-5^ | 0.85[0.38;1.32] | 3.90x10^-4^ |
| octanoylcarnitine | 1.45[0.91;1.99] | 1.58x10^-7^ | 1.66[0.98;2.33] | 1.88x10^-6^ | 0.66[0.3;1.01] | 2.61x10^-4^ | 0.78[0.34;1.22] | 5.77x10^-4^ |
| stearoylcarnitine | 1.22[0.68;1.77] | 1.06x10^-5^ | 1.38[0.7;2.06] | 6.80x10^-5^ | 0.78[0.43;1.14] | 1.80x10^-5^ | 0.84[0.4;1.28] | 1.91x10^-4^ |
| hexadecanedioate | 1.43[0.88;1.99] | 4.32x10^-7^ | 1.69[1;2.39] | 2.18x10^-6^ | 0.81[0.46;1.16] | 6.72x10^-6^ | 0.94[0.5;1.38] | 2.86x10^-5^ |
| tetradecanedioate | 1.77[1.19;2.35] | 2.94x10^-9^ | 2[1.25;2.76] | 2.25x10^-7^ | 0.86[0.49;1.23] | 5.18x10^-6^ | 1.06[0.59;1.53] | 1.02x10^-5^ |
| 10-heptadecenoate (17:1n7) | 1.32[0.78;1.86] | 1.92x10^-6^ | 1.55[0.85;2.25] | 1.52x10^-5^ | 0.84[0.48;1.2] | 5.99x10^-6^ | 1.01[0.56;1.47] | 1.50x10^-5^ |
| dihomo-linoleate (20:2n6) | 2.02[1.51;2.53] | 1.63x10^-14^ | 2.14[1.48;2.8] | 3.01x10^-10^ | 1.12[0.78;1.47] | 3.05x10^-10^ | 1.27[0.84;1.71] | 9.01x10^-9^ |
| nonadecanoate (19:0) | 1[0.49;1.51] | 1.26x10^-4^ | 1.07[0.42;1.73] | 1.33x10^-3^ | 0.7[0.35;1.05] | 8.36x10^-5^ | 0.77[0.33;1.21] | 5.79x10^-4^ |
| palmitate (16:0) | 1.87[1.35;2.39] | 3.65x10^-12^ | 2.03[1.35;2.72] | 7.59x10^-9^ | 1.01[0.65;1.38] | 4.60x10^-8^ | 1.16[0.7;1.61] | 7.07x10^-7^ |
| 5-dodecenoate (12:1n7) | 1.93[1.38;2.47] | 6.23e^-12^ | 2.2[1.52;2.88] | 2.75x10^-10^ | 1[0.65;1.35] | 2.50x10^-8^ | 1.14[0.71;1.58] | 2.97x10^-7^ |
| 4-androsten-3beta,17beta-diol disulfate 1* | 1.79[1.11;2.47] | 2.61x10^-7^ | 1.84[0.98;2.7] | 2.75x10^-5^ | 1.02[0.58;1.47] | 7.39x10^-6^ | 1.03[0.47;1.59] | 3.55x10^-4^ |
| cortisol | 1.7[1.17;2.24] | 4.99x10^-10^ | 1.9[1.19;2.61] | 2.06x10^-7^ | 0.83[0.47;1.18] | 5.86x10^-6^ | 1.02[0.56;1.48] | 1.41x10^-5^ |
| HWESASXX* | 0.92[0.37;1.47] | 1.08x10^-3^ | 1.29[0.58;1.99] | 3.99x10^-4^ | 0.69[0.33;1.05] | 1.72x10^-4^ | 0.8[0.35;1.26] | 6.06x10^-4^ |
| caffeine | 1.35[0.81;1.89] | 9.25x10^-7^ | 1.31[0.6;2.02] | 2.97x10^-4^ | 0.82[0.47;1.17] | 5.70x10^-6^ | 0.78[0.33;1.23] | 6.88x10^-4^ |

**the genotype score is calculated differently for SBP and DBP.

## Table S3. Blood pressure metabolite levels and risk of all-cause mortality in TwinsUK.

|  | Cox-Model 1 | | Cox-Model 2 | | Cox-Model 3 | |
| --- | --- | --- | --- | --- | --- | --- |
|  | **Overall sample**  **(186 deaths)** | | **no BP measure**  **(102 deaths)** | | **BP measure**  **(84 deaths)** | |
| **Metabolite** | **HR[95%CI]** | **P** | **HR[95%CI]** | **P** | **HR[95%CI]** | **P** |
| palmitate (16:0) | 1.13[0.78;1.63] | 0.51 | 0.89[0.54;1.49] | 0.66 | 1.6[0.92;2.8] | 0.10 |
| nonadecanoate (19:0) | 1.1[0.85;1.43] | 0.46 | 1.09[0.75;1.56] | 0.66 | 1.19[0.81;1.74] | 0.37 |
| dihomo-linoleate (20:2n6) | 0.66[0.48;0.89] | **0.01** | 0.63[0.41;0.96] | **0.03** | 0.67[0.42;1.08] | 0.1 |
| octanoylcarnitine | 0.97[0.8;1.17] | 0.73 | 1.08[0.83;1.42] | 0.56 | 0.85[0.64;1.13] | 0.27 |
| 5-dodecenoate (12:1n7) | 1.15[0.88;1.5] | 0.31 | 1.26[0.87;1.82] | 0.22 | 1.11[0.74;1.67] | 0.61 |
| 10-heptadecenoate (17:1n7) | 1.06[0.75;1.52] | 0.73 | 0.98[0.6;1.62] | 0.95 | 1.05[0.61;1.8] | 0.86 |
| stearoylcarnitine | 1.02[0.88;1.18] | 0.82 | 1.14[0.93;1.38] | 0.21 | 0.89[0.7;1.13] | 0.33 |
| tetradecanedioate | 0.76[0.56;1.03] | 0.08 | 0.73[0.48;1.11] | 0.14 | 0.75[0.48;1.17] | 0.21 |
| hexadecanedioate | 1.49[1.08;2.05] | **0.02** | 1.71[1.09;2.68] | **0.02** | 1.32[0.82;2.12] | 0.26 |
| lactate | 1.06[0.85;1.32] | 0.59 | 1.19[0.89;1.6] | 0.24 | 0.86[0.62;1.19] | 0.36 |
| caffeine | 0.83[0.71;0.96] | **0.01** | 0.83[0.68;1.02] | 0.07 | 0.81[0.64;1.02] | 0.07 |

Analyses adjusted for age, age^2^, BMI.

## Table S4. Replication results for hexadecanedioate in the KORA and Hertfordshire cohorts.

|  |  | **KORA (N=1494)** | | **Hertfordshire (N=1515)** | |
| --- | --- | --- | --- | --- | --- |
| **Phenotype** | **Metabolite** | **Beta[95%CI]** | **P** | **Beta[95%CI]** | **P** |
| SBP | hexadecanedioate | 1.42[0.37;2.47] | 0.01 | 1.58[0.56;2.60] | 0.02 |
| DBP | hexadecanedioate | 0.64[0.09;1.19] | 0.02 | 0.56[0.02;1.1 | 0.04 |

Analyses adjusted for sex, age, age^2^ and BMI.

## Table S5. BP - hexadecanedioate association in the TwinsUK cohort including those on treatment by adding 10/5mmHg to treated BP.

|  | **SBP** | | **DBP** | |
| --- | --- | --- | --- | --- |
| **Metabolite** | **Beta[95%CI]** | **P** | **Beta[95%CI]** | **P** |
| hexadecanedioate | 1.13[0.66;1.61] | 3.40x10^-6^ | 0.78[0.46;1.10] | 1.70x10^-6^ |

Analyses adjusted for age, age^2^, metabolite batch, BMI and family relatedness.

## Figure S1. Flowchart of the study design and analysis pipeline in the TwinsUK cohort.


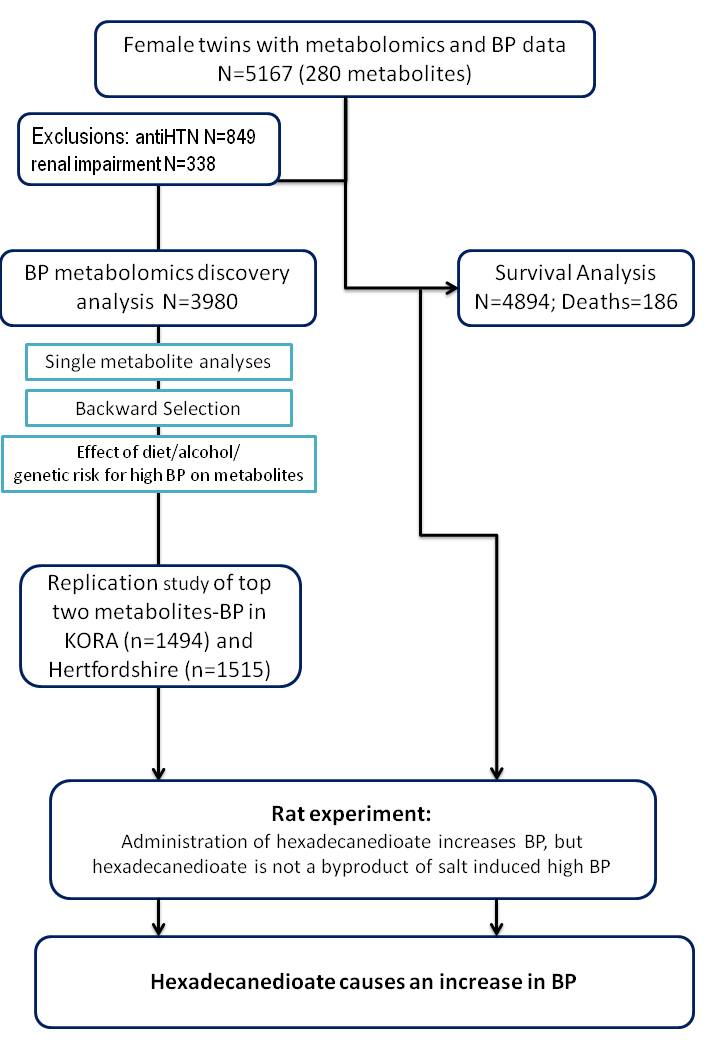


## Figure S2.Bivariate correlation between the 15 metabolites independently associated with BP

## Figure S3. Kaplan Meier plots of all-cause mortality for hexadecanedioate, dihomo-linoleate (20:2n6) and caffeine in TwinsUK.


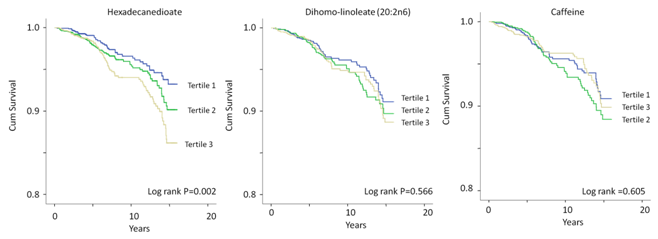

Supplement: Supplementary file 1 [file hyp-66-422-s001.docx]
